# Coexistent spontaneous coronary artery dissection and takotsubo syndrome: does one cause the other?

**DOI:** 10.1186/s43044-023-00413-x

**Published:** 2023-10-11

**Authors:** John E. Madias

**Affiliations:** 1 Icahn School of Medicine at Mount Sinai, New York, NY USA; 2 Division of Cardiology, Elmhurst Hospital Center, 79-01 Broadway, Elmhurst, NY 11373 USA

To the Editor,

I read with interest the paper by Saleemi et al., reporting on two women, 80- and 65-year old, who suffered both spontaneous coronary artery dissection (SCAD) and takotsubo syndrome (TTS) [1]. Although their gender and the emotional stress precipitants are in keeping with what is typical for both SCAD and TTS, their ages were atypical for SCAD, which usually is encountered in younger women [2], than the ones described herein [1]. In both patients SCAD affected the left anterior descending coronary artery, the vessel most often involved with SCAD [2]. In their second patient [1], the authors discuss the possibility that TTS could have been precipitated by the SCAD-induced chest pain; there is no much doubt that we will be witnessing more cases of simultaneous occurrence of SCAD and TTS in the future, and the authors’ point that it is important, when patients are diagnosed with TTS, that coronary angiograms are carefully scrutinized to exclude underlying SCAD [1] cannot be overemphasised. In addition, consideration should be given to the previously advanced hypothesis that TTS could potentially precipitate SCAD by an “excessive vigorous contraction of the left ventricle (LV) base in conjunction with the adjacent akinetic/dyskinetic systolic “ballooning” of the LV midmyocardium and apex”, which in predisposed individuals, “could form a prerequisite anatomic/functional substrate for the causation of SCAD [3]. Also, in exploring whether patients with diagnosed or suspected SCAD have also suffered a TTS, we should try, to implement early and frequently point of care ultrasound [4], following coronary angiography, to evaluate whether more patients with SCAD have also suffered TTS. Thus, pathophysiologically one should consider an amphidromic association between TTS and SCAD [1, 3, 5] at play.

## Data Availability

Not applicable.

## Abbreviations

LV: Left ventricle
SCAD: Spontaneous coronary artery dissection
TTS: Takotsubo syndrome
